# Radiomic signatures from T2W and DWI MRI are predictive of tumour hypoxia in colorectal liver metastases

**DOI:** 10.1186/s13244-023-01474-x

**Published:** 2023-07-21

**Authors:** Zuhir Bodalal, Nino Bogveradze, Leon C. ter Beek, Jose G. van den Berg, Joyce Sanders, Ingrid Hofland, Stefano Trebeschi, Kevin B. W. Groot Lipman, Koen Storck, Eun Kyoung Hong, Natalya Lebedyeva, Monique Maas, Regina G. H. Beets-Tan, Fernando M. Gomez, Ieva Kurilova

**Affiliations:** 1 Department of Radiology, The Netherlands Cancer Institute, Plesmanlaan 121, 1066 CX Amsterdam, The Netherlands; 2 GROW School for Oncology and Developmental Biology, Maastricht University, Maastricht, The Netherlands; 3 Department of Radiology, American Hospital Tbilisi, Tbilisi, Georgia; 4 Department of Medical Physics, The Netherlands Cancer Institute, Amsterdam, The Netherlands; 5 Department of Pathology, The Netherlands Cancer Institute, Amsterdam, The Netherlands; 6 Core Facility Molecular Pathology & Biobank, The Netherlands Cancer Institute, Amsterdam, The Netherlands; 7 Hospital Clinic-Hospital Sant Joan de Deu, Barcelona, Spain

**Keywords:** Colorectal cancer, Colorectal liver metastasis, Hypoxia, MRI, Radiomics

## Abstract

**Background:**

Tumour hypoxia is a negative predictive and prognostic biomarker in colorectal cancer typically assessed by invasive sampling methods, which suffer from many shortcomings. This retrospective proof-of-principle study explores the potential of MRI-derived imaging markers in predicting tumour hypoxia non-invasively in patients with colorectal liver metastases (CLM).

**Methods:**

A single-centre cohort of 146 CLMs from 112 patients were segmented on preoperative T2-weighted (T2W) images and diffusion-weighted imaging (DWI). HIF-1 alpha immunohistochemical staining index (high/low) was used as a reference standard. Radiomic features were extracted, and machine learning approaches were implemented to predict the degree of histopathological tumour hypoxia.

**Results:**

Radiomic signatures from DWI b200 (AUC = 0.79, 95% CI 0.61–0.93, *p* = 0.002) and ADC (AUC = 0.72, 95% CI 0.50–0.90, *p* = 0.019) were significantly predictive of tumour hypoxia. Morphological T2W TE75 (AUC = 0.64, 95% CI 0.42–0.82, *p* = 0.092) and functional DWI b0 (AUC = 0.66, 95% CI 0.46–0.84, *p* = 0.069) and b800 (AUC = 0.64, 95% CI 0.44–0.82, *p* = 0.071) images also provided predictive information. T2W TE300 (AUC = 0.57, 95% CI 0.33–0.78, *p* = 0.312) and b = 10 (AUC = 0.53, 95% CI 0.33–0.74, *p* = 0.415) images were not predictive of tumour hypoxia**.**

**Conclusions:**

T2W and DWI sequences encode information predictive of tumour hypoxia. Prospective multicentre studies could help develop and validate robust non-invasive hypoxia-detection algorithms.

**Critical relevance statement:**

Hypoxia is a negative prognostic biomarker in colorectal cancer. Hypoxia is usually assessed by invasive sampling methods. This proof-of-principle retrospective study explores the role of AI-based MRI-derived imaging biomarkers in non-invasively predicting tumour hypoxia in patients with colorectal liver metastases (CLM).

**Graphical Abstract:**

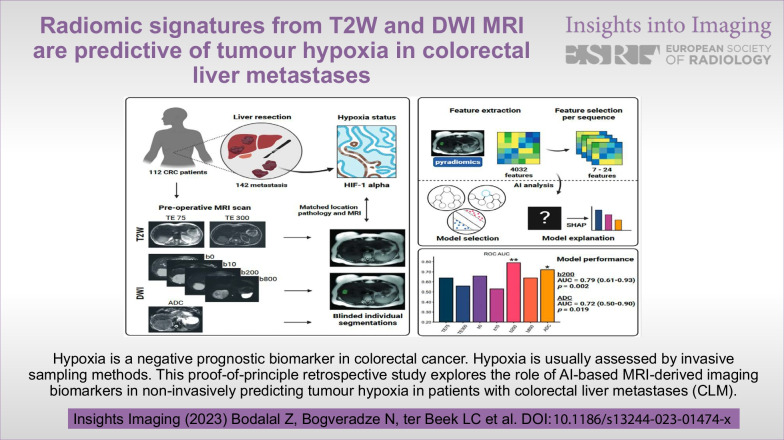

**Supplementary Information:**

The online version contains supplementary material available at 10.1186/s13244-023-01474-x.

## Key points

- Tumour hypoxia is a valuable prognostic/predictive biomarker in colorectal cancer.
- This proof-of-principle study demonstrates non-invasive associations between MR-radiomics and tumour hypoxia.
- Radiomics from DWI b200, ADC, and T2W TE75 predicted tumour hypoxia.

**Supplementary Information:**

The online version contains supplementary material available at 10.1186/s13244-023-01474-x.

## Introduction

Tumour hypoxia is a valuable prognostic and predictive biomarker in colorectal cancer (CRC) [1–4]. Genome-wide and microRNA analyses have shown that CRC tumours with a hypoxic microenvironment showed significantly worsened clinical outcomes, especially disease-free survival [5, 6]. Resistance to chemotherapy, radiotherapy, and immunotherapy has been associated with tumour hypoxia in other solid tumours [7–13]. Furthermore, when evaluating the role of hypoxia on local therapies such as percutaneous ablation, transarterial chemotherapy, and radioembolisation, several studies have found that it also negatively impacts resistance to the treatment and/or induces a more aggressive clonal cell selection [14–17]. Hypoxia-driven elevation of proangiogenic factors is a hallmark of colorectal cancer and its liver metastasis [18]. Specifically, in the treatment of colorectal liver metastases, the hypoxic status has also been shown to influence the resistance to antiangiogenic drugs [19] or their susceptibility regarding radiation therapy, thereby impacting the dosimetric planning for Y-90 radioembolisation [20]. Moreover, in the era of immune therapies, hypoxia has also been revealed to play a major role in the current understanding of the immune microenvironment of colorectal liver metastases [21].

Polarographic electrodes inserted directly into the tumour are considered the gold standard for measuring tumour hypoxia. However, in the routine clinical workflow, histopathological analysis is more commonly performed, with tissue hypoxia markers, such as hypoxia-inducible factor-1 (HIF-1) alpha, being the most relevant [22]. Both approaches suffer from similar shortcomings: invasiveness, limitation to accessible tumours, and inability to take tumour heterogeneity into account [23]. Moreover, these methods cannot provide longitudinal information on changes in the oxygenation of the microenvironment. Developing a non-invasive, robust imaging-based technique to assess tumour hypoxia would improve patient selection, treatment monitoring, and treatment modification.

As medical image analysis research has gained recognition in the clinical world, increasingly relevant applications of radiomics coupled with machine learning have emerged. Radiomic features and signatures have been associated with long-term prognosis and response to local and systemic therapy [24–28]. Prominent among the use cases for radiomics has been the domain of radiogenomics, where morphological phenotypes are linked to the underlying tumour genotype [29, 30]. As more information is gathered from tumours, the scope of radiogenomics has expanded beyond strict somatic mutation analysis and now encompasses broader biological parameters, particularly from the tumour microenvironment [30, 31].

Past literature has attempted to perform histological/radiological correlation using radiomics. More specifically, CT-derived radiomic features have been associated with PET imaging of hypoxia [32]. Other studies have shown links between radiomics-based patient stratification (e.g. on response or survival) and gene signatures related to the hypoxia pathway [33–35].

In this proof-of-principle project, we aimed to use MRI-derived radiomic features and novel machine learning approaches to non-invasively predict the degree of histopathological tumour hypoxia in patients with colorectal liver metastases. We hypothesise that by leveraging medical image analysis techniques and ubiquitous imaging modalities, non-invasive insight into CRC hypoxia can be obtained.

## Methods

### Patient cohort and data collection

**Fig. 1:**
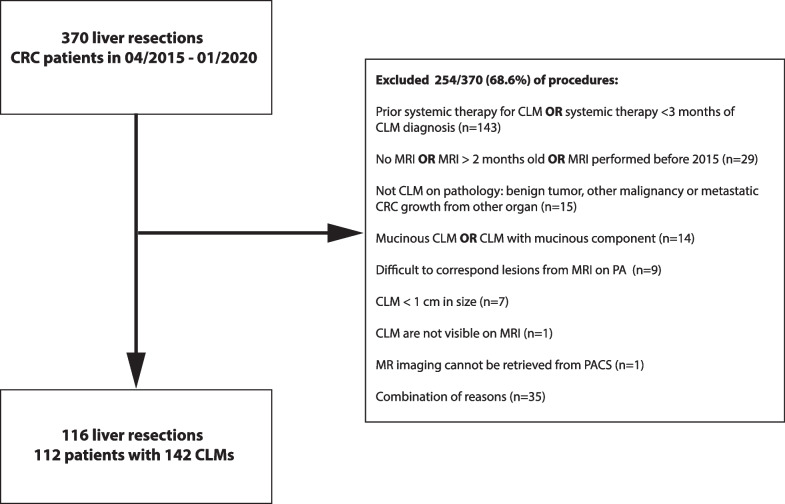
Study exclusion criteria. CRC-colorectal cancer, CLM-colorectal liver metastases, PA-pathology

We retrospectively collected clinical patient data from all colorectal cancer patients with known liver metastases who had undergone liver resection at our institution (The Netherlands Cancer Institute, Amsterdam) between April 2015 and January 2020. Our initial cohort comprised 370 patients, from which 248 subjects were excluded for prior systemic therapy (*n* = 143), missing or old MRIs (> 2 months, *n* = 29), non-cancerous liver lesions (*n* = 15) and mucinous subtype (*n* = 14). Patients that had received prior systemic therapy were excluded due to the expected impact the different treatments would have on the microenvironment, introducing noise and variability. Cases where accurate matching between imaging and pathology was not possible were immediately excluded (*n* = 9). Reasons for failed matching included the presence of multiple lesions within the same liver segment or a discrepancy in liver segments reported between the pathology and MRI report. Figure 1 depicts a detailed flowchart highlighting cases that were excluded. The final cohort used in this analysis was 112 patients with 116 procedures and 142 metastases for whom both MRI data and histopathological tumour hypoxia information were collected. All research performed has received bioethical approval from the Institutional Review Board (IRB) of the The Netherlands Cancer Institute, Amsterdam (IRBdm20-066).

### MRI and tumour segmentation

The Picture Archive and Communication System (PACS) was retrospectively queried to retrieve the preoperative MRI scans for the patients in our cohort. For each liver resection, we selected the MRI scan closest to the date before the procedure. Apart from the echo time, two T2-weighted sequences were used (TE75, TE300) with a field-of-view of 400 × 400 × 233 (AP × RL × FH) mm, a reconstructed in-plane voxel of 1 × 1 (AP × RL) mm, slice thickness of 5 mm FH, echo times of 75 and 300 ms respectively and a repetition time of 2725 ms, without fat suppression. Five routinely available sequences were collected from the diffusion-weighted scan (b0, b10, b200, b800, and ADC) with a field-of-view of 450 × 400 × 251 (AP × RL × FH) mm, reconstructed in-plane voxel size of 1.75 × 1.75 (AP × RL) mm, echo time of 86 ms, repetition time of 1447 ms, and a SPAIR fat suppression pulse was used—leading to a maximum of seven sequences available per liver resection. The selected echo times and b-values were based on the standard MRI protocols at our centre for the evaluation of colorectal liver metastases, established through a combination of clinical practice guidelines and the need to balance image quality and acquisition time, in accordance with the preferences of our institution's radiology department. All four b-value sequences plus ADC were collected to explore all possible associations between diffusion-weighted imaging and hypoxia. The scans were acquired on a 3 T Philips Achieva and a 3 T Philips Ingenia (Philips Healthcare, Best, the Netherlands), with an anterior and built-in posterior coil as receiver coils. Gadolinium-containing contrast agents used in our liver examination were administered after the included T2W and DWI sequences were acquired. 3DSlicer (v.4.10.2) was used to view the radiological images and perform the manual segmentation (N.B., board-certified radiologist with four years of expertise in liver imaging). As a single radiologist performed all the segmentations, we were able to mitigate the risk of inter-reader variability. Each sequence was segmented independently. No assumptions were made regarding the possibility of generalising segmentations between sequences. Additional file 1: S1 shows the pairwise correlation between the segmentations per sequence in terms of three dimensional diameter and volume.

Similar to how the two largest lesions (when present) were resected and subsequently selected for staining, an expert radiologist applied the same criteria to CLM delineation on MRI images. Liver metastases were matched between MRI and pathology based on lesion location. Segmentations of the two largest liver metastases were performed to account for inter-tumoural heterogeneity. Pairwise analysis was performed to assess the degree of correlation between the segmentations of different sequences (in terms of volume and diameter, Additional file 1: S1). All image and annotation files were saved according to the NRRD format.

### Radiomic feature extraction and selection

From each delineated lesion, 4032 radiomics features were extracted using PyRadiomics (v3.0). The extracted features included first-order statistics, two-dimensional and three-dimensional shape features, grey level dependence matrix (GLDM) features, grey level run length matrix (GLRLM) features, grey level co-occurrence matrix (GLCM) features, grey level size zone matrix (GLSZM) features, and neighboring grey tone difference (NGTDM) matrix features, each with a set of filters applied to them (square, square root, logarithm, exponential, gradient, wavelet, Laplacian of Gaussian, and three-dimensional local binary pattern). The dataset was normalised by centering the data at the mean with standard deviation to account for potential scanner level differences. Each radiomic feature vector represented the morphological phenotype of the segmented region of interest. A complete description of the radiomic features generated by PyRadiomics can be found in van Griethuysen et al. [36].

The resulting feature space suffered from the curse of dimensionality, where the number of features was greater than the samples. This situation typically results in increased noise in the data and makes the machine learning model more prone to overfitting the training data and less likely to generalise to unseen information. We performed ensemble feature selection to identify the most representative/relevant features within the sizable radiomic feature space. In this method, seven supervised feature selection approaches (Chi-square, correlation with outcome, random forest, linear regression, logistic regression, recursive feature selection, and light gradient boost machine) would independently select the top 100 most relevant features for the specific outcome (e.g. hypoxia staining index high vs. low). Subsequently, only the features chosen by the majority of methods (> = 4/7 approaches) would be included in the analysis. Ensemble feature selection approaches help ensure that only the features with the maximum relevance would be used in the study by leveraging the strengths of specific selection methods to overcome weaknesses in others [37, 38]. The feature selection was performed exclusively on the training set to prevent data leakage. The selected features were then generalised to the independent test set. The selected features per sequence can be seen in Additional file 1: S2.

### Histopathological assessment of tumour hypoxia

Our institutional biobank was queried to obtain archival resection specimens for the patients included in the final cohort. As a critical regulator of cellular response to hypoxia, HIF-1 alpha is a reliable and permanent histopathological marker of tumour hypoxia in colorectal cancer, which can be detected accurately after tissue excision and storage [39]. Tumour hypoxia was assessed by a consensus reading of two board-certified pathologists based on HIF-1 alpha immunohistochemical staining of each patient’s resection samples (Additional file 1: S3). When multiple liver lesions were resected, specimens from the two largest liver metastases were stained. The pathologists selected two representative colorectal liver metastases slides (centre and periphery) for each tumour. HIF-1 alpha expression was classified based on an established semi-quantitative (and subjective) staining index [40, 41]. The staining index (range: 0–9) was determined by multiplying the score for the intensity of the staining (none = 0, weak = 1, moderate = 2, strong = 3) with the score of the proportion of positive HIF-1 alpha cells (< 10% = 1, 10–50% = 2, > 50% = 3) that were both qualitatively assessed in consensus reading with both pathologists. In our cohort, the indices ranged from 0 to 6, with no cases having a score = 3 and a score = 5 being impossible due to the multiplication process. The staining index cut-off value was set as 0–2 for negative/low HIF-1 alpha expression and 3–6 for high expression, coinciding with the median [40]. As our pathologists deemed that approach more clinically relevant, the slide with a higher percentage of positive cells and stronger staining intensity was used to calculate the staining index.

### Modeling and machine learning

The analysis was performed on data from 116 resections consisting of a reference standard on pathology (HIF-1 staining index, high vs. low) paired with a pre-resection MRI scan (with the available sequences). This data was divided into a training set (75%) to train and optimise the machine learning algorithm and an independent test set (25%) to evaluate the model’s ability to generalise to unseen data. To discover the best machine learning pipeline (composed of a classifier, hyperparameters, and processing steps) to model the data, a tree-based evolutionary algorithm was used (via the Python library, TPOT) [42]. We supplied the training data to the genetic algorithm in this setup, and the machine learning space was explored automatically using a genetic selection approach. In each generation, candidate pipelines are evaluated using a five-fold cross-validation scheme. The top-performing models are then automatically “mutated” in the sense of being modified to increase performance in the next round. This model creation and evaluation process continues until a final candidate machine learning algorithm emerges. The selected model and hyperparameters were tested on the unseen independent test set (Additional file 1: S4).

### Statistical analysis

To assess predictive performance, we measured the area under the receiver operating curve (AUC). Additionally, we computed precision-recall AUC (PR AUC), accuracy, sensitivity, specificity, the F1 score, the negative predictive value (NPV), and the positive predictive value (PPV). Bootstrapping with 1000 samples was performed to compute 95% confidence intervals for all the evaluation metrics. Mann–Whitney U test was used to compare the predictions between the classes and test for significance [27]. *p* values above the conventional threshold (0.05) were considered to be statistically significant.

**Fig. 2:**
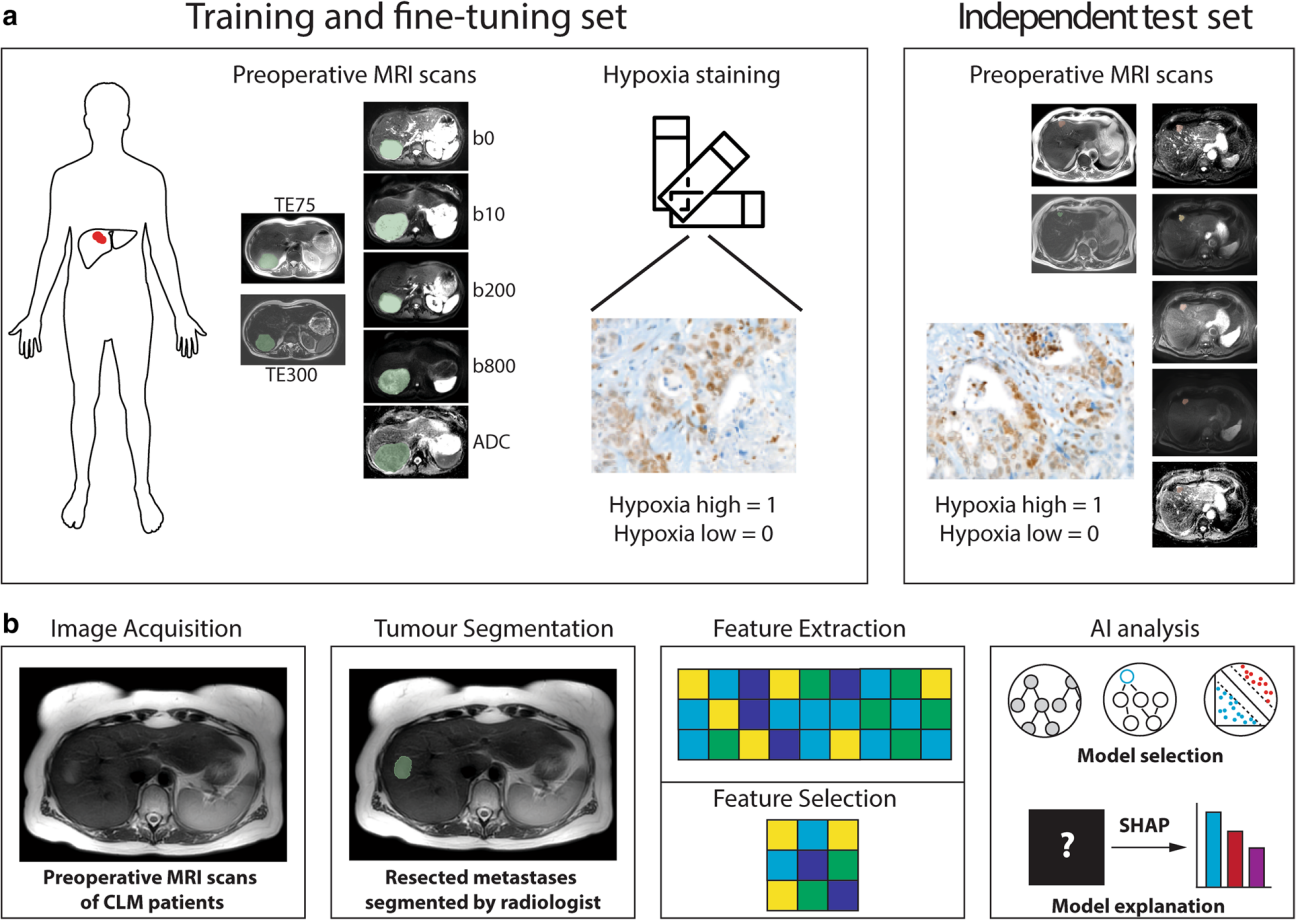
An illustrative diagram highlighting the (**a**) study design as well as the (**b**) radiomic/machine learning workflows. Expert readers delineated pre-resection MRI images, and from these regions of interest, handcrafted radiomic features are extracted. Machine learning classifier selection, training, and hyperparameter optimisation were performed using an evolutionary genetic approach. The candidate model is then evaluated on its predictive performance with an unseen validation cohort

Shapley values were calculated for the features used by the machine learning models to gain insight into the AI predictions on the unseen test set. The features with the most impact on the prediction were plotted for each MRI sequence. Figure 2 gives a broad overview of the study design and analysis workflow. All analyses were performed using Python 3.6 (scikit 0.22.1, pandas 1.0.1, numpy 1.18.1, matplotlib 3.1.3, tpot 0.11.2, shap 0.37.0).

## Results

### Clinical and imaging characteristics

**Fig. 3:**
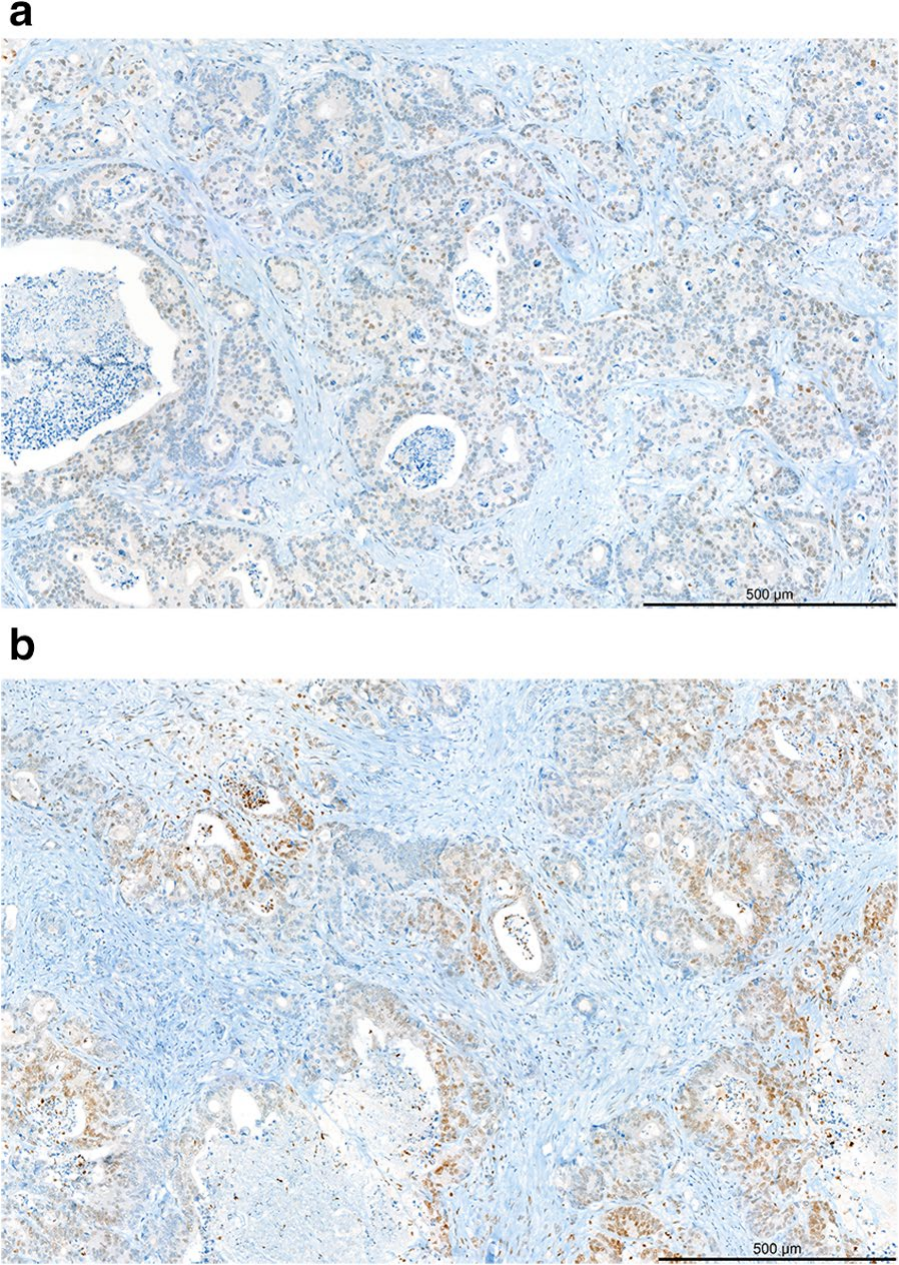
HIF-1 alpha staining. HIF-1 alpha staining in colorectal liver metastases. Upper panel: weak nuclear staining for HIF-1 alpha in a minority of tumour cells (10%). Lower panel: strong nuclear staining for HIF-1 alpha in a proportion of tumour cells (30%). Scale bar 500 µm

A final cohort of 112 patients across 116 liver resections with 142 colorectal liver metastases was included in our analysis (Fig. 1). Four patients underwent resection twice at different points in their treatment history, resulting in a total of 116 resections with both imaging and biological data. The average age of our patients was 64.1 years (± 10), with a slight preference for males (*n* = 68, 59%). All patients in our dataset underwent liver resection. In cases where only a single lesion was resected (*n* = 86 patients, 77%), that lesion would be directly included in the study. In patients where > 1 lesion was resected (*n* = 26 patients, 23%), the largest two lesions > 1 cm were included. Median time gap between the pre-resection MRI acquisition and liver resection was 25.5 days (IQR = 16.5–35 days). HIF-1 alpha staining was performed on the archival specimens from the NKI Core Facility—Molecular Pathology and Biobank (staining protocol Additional file 1: S3). Based on the hypoxia staining intensity index score, patients were labeled as “hypoxia low” (*n* = 67, 47%) or “hypoxia high” (*n* = 75, 53%) (Fig. 3). As the two classes (hypoxia high/low) were balanced, no upsampling/undersampling technique was used.

Pre-resection MRI scans were then delineated by a radiologist focusing on T2W TE75 (available for *n* = 92/116, 79%), T2W TE300 (*n* = 85/116, 73%), DWI b0 (*n* = 100/116, 86%), DWI b10 (*n* = 113/116, 97%), DWI b200 (*n* = 99/116, 85%), DWI b800 (*n* = 116/116, 100%), and ADC (*n* = 110/116, 95%), when available. Detailed characterisation of our patient cohort can be found in Table 1. Each sequence was segmented individually and correlation between the segmentations of the sequences was found to be > 0.95 (for diameter) and > 0.97 (for volume). Detailed pairwise correlations between the segmentations of each sequence can be seen in Additional file 1: S1.

**Table 1:** Patient characteristics

Median age (median, IQR)	65	(57–71)
*Gender (n, %)*		
Male	68	59%
Female	48	41%
Overall survival in months (median, IQR)	23.6	(11.3–34.1)
Tumour size in cm, pathology (median, IQR)	2.5	(1.8–3.5)
Tumour size in cm, on MRI (median, IQR)	2.1	(1.7–3.3)
*Hypoxia staining score (n lesions, %)*		
Low	67	47%
High	75	53%
*Hypoxia staining index score* *(n resections, %)*		
Score = 0	20	14%
Score = 1	23	16%
Score = 2	24	17%
Score = 3	-	-
Score = 4	55	39%
Score = 5	-	-
Score = 6	20	14%
*Location of tumours (n lesions, %)*		
S1	1	1%
S2	21	11%
S3	29	15%
S4	23	12%
S5	25	13%
S6	34	17%
S7	34	17%
S8	33	17%
*Availability of sequence (n resections, %)*		
T2W TE75	92	79%
T2W TE300	85	73%
b0	100	86%
b10	113	97%
b200	99	85%
b800	116	100%
ADC	110	95%

### Radiomic analysis

Feature extraction from each of the seven MRI sequences yielded 4032 radiomic features encoding the full morphological phenotype of each segmented liver metastasis. Supervised ensemble feature selection identified radiomic signatures per MRI sequence most relevant for predicting tumour hypoxia. These signatures ranged between 7 and 24 radiomic features based on the MR sequence (*see* Additional file 1: S2). For each radiomic MRI signature, we used an evolutionary tree-based algorithm approach to select, train, and optimise the hyperparameters of a machine learning algorithm to predict hypoxia (Additional file 1: S4).

Radiomic signatures from DWI b200 (AUC = 0.79, 95% CI = 0.61–0.93, *p* = 0.002) and ADC (AUC = 0.72, 95% CI = 0.50–0.90, *p* = 0.019) were significantly predictive of tumour hypoxia. Anatomical imaging in the form of T2W TE75 (AUC = 0.64, 95% CI 0.42–0.82, *p* = 0.092) and functional b0 (AUC = 0.66, 95% CI 0.46–0.84, *p* = 0.069) and b800 (AUC = 0.64, 95% CI 0.44–0.82, *p* = 0.071) images showed trends toward predictive ability. Although the *p* values did not reach the conventional threshold for statistical significance, they are close to this threshold, suggesting that there may be some predictive power associated with these imaging sequences, particularly in the context of our modest dataset.

**Fig. 4:**
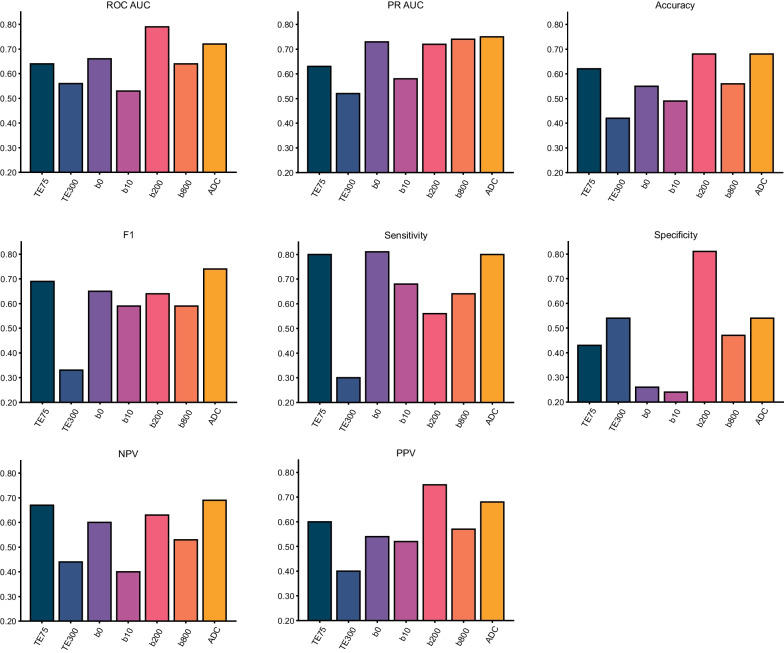
Performance metrics of the machine learning algorithms that were selected for each of the MRI sequences. NPV, negative predictive value; PPV, positive predictive value; PR AUC, precision-recall AUC

In our cohort, radiomic features derived from T2W TE300 (AUC = 0.57, 95% CI 0.33–0.78, *p* = 0.312) and b10 (AUC = 0.53, 95% CI 0.33–0.74, *p* = 0.415) images were poorly predictive of tumour hypoxia (Fig. 4). Full performance metrics of each algorithm on its respective MRI sequence can be found in Table 2.

**Table 2:** Performance metrics of the machine learning algorithms

	ROC AUC	PR AUC	Accuracy	Sensitivity	Specificity	NPV	PPV	F1 score	*p* value
TE75	0.64 (0.42–0.82)	0.63 (0.40–0.83)	0.62 (0.45–0.79)	0.80 (0.57–1.00)	0.43 (0.15–0.71)	0.67 (0.30–1.00)	0.60 (0.37–0.80)	0.69 (0.48–0.83)	0.092
TE300	0.57 (0.33–0.78)	0.52 (0.28–0.70)	0.42 (0.23–0.62)	0.30 (0.08–0.58)	0.54 (0.27–0.80)	0.44 (0.18–0.69)	0.40 (0.10–0.73)	0.33 (0.10–0.56)	0.312
b0	0.66 (0.46–0.84)	0.73 (0.48–0.89)	0.55 (0.39–0.71)	0.81 (0.60–1.00)	0.26 (0.07–0.50)	0.60 (0.17–1.00)	0.54 (0.35–0.74)	0.65 (0.44–0.81)	0.069
b10	0.53 (0.33–0.74)	0.58 (0.37–0.79)	0.49 (0.31–0.66)	0.68 (0.47–0.89)	0.24 (0.06–0.47)	0.40 (0.09–0.71)	0.52 (0.32–0.71)	0.59 (0.40–0.75)	0.415
b200	0.79 (0.61–0.93)	0.72 (0.51–0.91)	0.68 (0.52–0.84)	0.56 (0.31–0.81)	0.81 (0.57–1.00)	0.63 (0.41–0.84)	0.75 (0.50–1.00)	0.64 (0.40–0.82)	**0.002**
b800	0.64 (0.44–0.82)	0.74 (0.55–0.88)	0.56 (0.42–0.72)	0.64 (0.40–0.83)	0.47 (0.23–0.71)	0.53 (0.27–0.79)	0.57 (0.37–0.77)	0.59 (0.40–0.76)	0.071
ADC	0.72 (0.50–0.90)	0.75 (0.54–0.94)	0.68 (0.53–0.82)	0.80 (0.60–0.95)	0.54 (0.27–0.78)	0.69 (0.36–0.91)	0.68 (0.50–0.87)	0.74 (0.56–0.87)	**0.019**

**Fig. 5:**
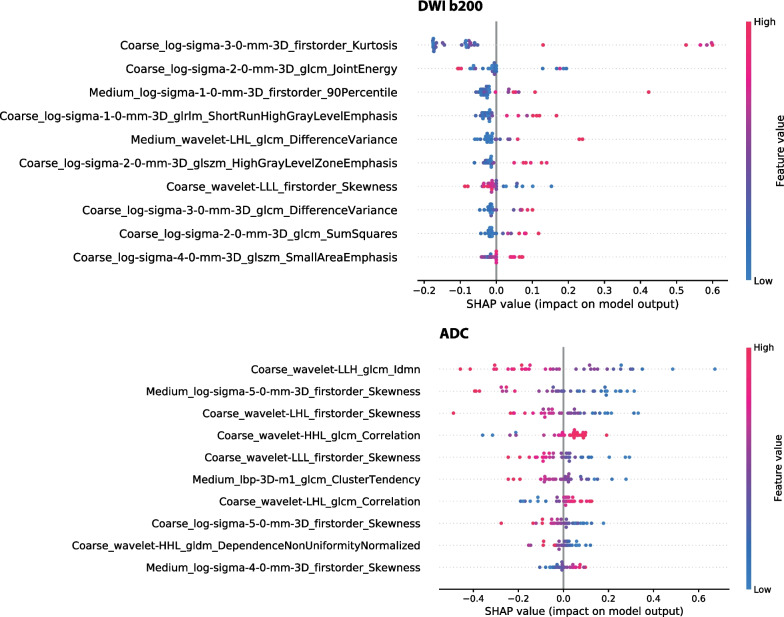
Shapley summary plots of the most relevant features for the prediction of tumour hypoxia in b200 and ADC. The SHAP value reflects the impact of the individual features on the prediction while the colour of the dots represents the value of that particular feature

Shapley values were computed for each of the machine learning algorithms to determine the impact of each feature on the prediction and their relative importance (Fig. 5). In the case of the two sequences where the AI model made statistically significant predictions, DWI b200 and ADC, the features within the radiomic signatures for hypoxia reflected morphological heterogeneity. On b200 images, the AI model relied on higher values of morphological heterogeneity to predict hypoxia. Conversely, a lower degree of heterogeneity was associated with hypoxia on ADC.

## Discussion

In this proof-of-concept study, we aimed to explore the potential of MRI-derived radiomic features in the non-invasive prediction of tumour hypoxia. Among the routinely-performed magnetic resonance imaging (MRI) scans in the clinical workflow, T2-weighted imaging and DWI have the most profound biological connection to hypoxia [43–45]. Preclinical studies have also shown that ADC and T2 mapping were helpful in imaging tumour hypoxia in murine models [46, 47]. With this in mind, we decided to focus the scope of our imaging study on T2W and DWI sequences and their derived parameter maps.

In our cohort of baseline colorectal liver metastases, radiomic signatures derived from the ADC map and DWI b200 image were significantly associated with histopathological tumour hypoxia. Imaging markers derived from T2W TE75, DWI b0, and DWI b800 were also predictive of the HIF-1 alpha staining index, albeit narrowly short of statistical significance. In general, lower spin echo times and higher b-value images were able to encode more information regarding the hypoxia status. These findings are in line with our current understanding of hypoxia under MRI.

Tumour hypoxia is generally influenced by perfusion and diffusion, with the former being related to *acute* hypoxia and the latter being associated with *chronic* hypoxia [48, 49]. As tissues rapidly grow, cellularity increases, a possible mechanism of water diffusion restriction. However, as tumour growth outpaces its vascularisation, hypoxia ensues, often resulting in necrotic areas with lower cellular density [50]. The ebb and flow between cell growth/density and vascularisation/hypoxia may be a mechanism for detecting hypoxic changes in functional DWI (and ADC). Diffusion is visualised primarily in the higher b-value sequences, outside the perfusion fraction range, and in the ADC map. In chronic tumour hypoxia, more information should be available from such images (i.e. b200, b800, and ADC).

Alongside diffusion-weighted functional sequences, T2W imaging can also be linked to hypoxia. During acquisition, the balance between oxygen-related paramagnetic molecules influences the T2 time constant of nearby tissue. The presence of electrons defines such molecules. Prominent examples include molecular oxygen, deoxyhaemoglobin, methaemoglobin and oxygen-related diamagnetic molecules such as oxyhaemoglobin. The higher the concentration of paramagnetic molecules, the shorter the T2 time constant [51, 52]. T2 images acquired under lower echo times yielded more useful information for the model to predict hypoxia. In our cohort, we observed that liver metastases were poorly visible on TE300, possibly resulting in lower-quality, less predictive radiomic features. The reduction of contrast-to-noise ratios among different tissues at higher echo times (due to T2 relaxation) could explain this phenomenon. In this case, the signal intensities of the various tissues would approach each other while decaying into the noise.

We observed that the radiomic features selected for each sequence collectively encoded morphological heterogeneity (Additional file 1: S2). The radiomic signatures for the DWI b200 and ADC models consisted of radiomic features derived at both coarse and medium resolutions. The radiomic signature for the ADC model, in particular, selected multiple features representing skewness (with various resolutions/filters). SHAP analysis on the model trained using DWI b200 data showed that higher levels of morphological heterogeneity generally influenced the model to predict hypoxia (except for Coarse_wavelet-LLL_firstorder_Skewness). The reverse was true for the ADC map, where lower morphological heterogeneity values were linked with tumour hypoxia prediction. Coarse_wavelet-LLL_firstorder_Skewness was selected as an impactful feature in both the b200 and ADC algorithms, further highlighting the potential for skewness as an MR imaging marker of hypoxia.

Most attempts at unlocking hypoxia information using imaging have focused on using PET. Tracers targeting HX4, nitroimidazole analogues, and ATSM have all been shown to visualise hypoxia [53–56]. The challenge with PET hypoxia imaging is that tumours with low oxygenation tend to be hypoperfused. Limited vascularisation and perfusion restrict the delivery of the PET tracer to the tumour (microenvironment) [57]. Radiomic features derived from non-targeted (anatomical and functional) imaging would help mitigate this shortcoming. Literature on the association between radiomics and tumour hypoxia, in general, is scarce, let alone specifically for colorectal cancer. Sanduleanu et al. developed and validated disease-agnostic radiomic signatures derived from FDG-PET and CT images [32]. The predictive performance of the PET/CT signatures was similar to those we identified in DWI/T2W imaging (AUC ranges = 0.7–8). In a small cohort of cholangiocarcinoma patients, Sadot et al. found associations between CT texture analysis and VEGF [58]. CT radiomic features were also used in histopathological correlation with pimonidazole, a nitroimidazole analogue hypoxia marker [59]. DCE MR imaging of 17 primary liver cancer patients also showed changes induced by anti-angiogenic therapy associated with hypoxia [60].

To our knowledge, this study is the first to explore associations between tumour hypoxia and radiomic features derived from routine MR sequences. Our dataset was retrospective in nature and had a modest cohort size due to the fact that HIF-1 alpha hypoxia staining is not part of the routine clinical workflow. As such, our reference standard had to be actively generated using archival specimens in collaboration with our pathology department. All hypoxia scores were generated from hypoxia staining of patient archival tissue performed exclusively for this project. Given the scarcity of suitable human tissue and the associated costs, the sample size of this study remained relatively modest. Since our ground truth is not routinely generated, creating large training or external datasets is challenging. Moreover, as the dataset is retrospectively derived, not all patients had all sequences available in the PACS, owing to the intricacies of real-world clinical data (e.g. referring physician preferences, timing, and patient-specific factors). Exploration of association with survival was also not possible due to the heterogeneity of this modestly-sized retrospective cohort. Patients whose baseline tissue and MR scans were included in this study would later receive different treatments making the comparison challenging, especially since the hypoxia status is derived from a lesion while the survival can be confounded by other patient-level differences (e.g., stage, comorbidities, etc.). Prospective studies with detailed patient inclusion and matching could explore the potential of hypoxia AI models to predict survival.

Notwithstanding these limitations, we could identify promising radiomic signatures for tumour hypoxia on T2W and DWI, especially on b200 and ADC. In clinical practice, the results of this study could open the gates for further research aimed at improving patient selection for various liver-directed therapies and prompt treatment modification by more aggressive tumour targeting or by the use of hypoxia-modifying drugs.

## Conclusions

Morphological phenotypes, as quantified by radiomic signatures, were found to predict tumour hypoxia. These findings suggest that anatomical T2W and functional DWI MRI sequences hold information that can be used for the non-invasive prediction of tumour hypoxia. Radiogenomic prediction of microenvironmental characteristics such as hypoxia could add significant insight into tumour biology, especially in a longitudinal setting. However, as this study was proof of concept, large-scale prospective multicentre studies would be needed to develop and validate robust hypoxia predictive algorithms.

## Supplementary Information

**Additional file 1**. Supplementary material containing the correlation between sequences, radiomic signatures, staining protocol, and selected model/hyperparameters.

## Data Availability

The datasets generated and/or analysed during the current study are not publicly available due to patient privacy regulations but are available from the corresponding author on reasonable request.

## Abbreviations

ADC: Apparent diffusion coefficient
AI: Artificial intelligence
ATSM: *N*-Methylthiosemicarbazone
AUC: Area under the curve
CI: Confidence interval
CLM: Colorectal liver metastases
CRC: Colorectal cancer
CT: Computed tomography
DCE MRI: Dynamic contrast-enhanced magnetic resonance imaging
DWI: Diffusion-weighted imaging
GLCM features: Grey level co-occurrence matrix features
GLDM features: Grey level dependence matrix features
GLRLM features: Grey level run length matrix features
GLSZM features: Grey level size zone matrix features
HIF-1 alpha: Hypoxia-inducible factor 1-alpha
HX4: Flortanidazole
IRB: Institutional Review Board
MRI: Magnetic resonance imaging
NGTDM features: Neighboring grey tone difference matrix features
NRRD format: Nearly raw raster data format
PACS: Picture archive and communication system
PET: Positron emission tomography
SHAP: SHapley Additive exPlanations
SPAIR: SPectral attenuated inversion recovery
T2W imaging: T2-weighted imaging
TPOT: Tree-based pipeline optimisation tool
VEGF: Vascular endothelial growth factor
