# Supplementary material for: Radiomic signatures from T2W and DWI MRI are predictive of tumour hypoxia in colorectal liver metastases
Source: Insights Imaging. 2023 Jul 21;14:133. doi: 10.1186/s13244-023-01474-x (PMC10361926; doi:10.1186/s13244-023-01474-x)
Supplement: Supplementary file 1 — Additional file 1. Supplementary material containing the correlation between sequences, radiomic signatures, staining protocol, and selected model/hyperparameters. [file 13244_2023_1474_MOESM1_ESM.pdf]

Radiomic signatures from T2W and DWI MRI are predictive of tumour hypoxia in colorectal liver metastases

ELECTRONIC SUPPLEMENTARY MATERIAL

S1. Correlation of volume and diameter between the segmentations of each sequence.

Volume correlation

	TE75 Volume	TE300 Volume	b0 Volume	b10 Volume	b200 Volume	b800 Volume	ADC Volume
**TE75 Volume**	1.0000	0.9797	0.9947	0.9917	0.9929	0.9939	0.9938
**TE300 Volume**	0.9797	1.0000	0.9836	0.9852	0.9811	0.9841	0.9846
**b0 Volume**	0.9947	0.9836	1.0000	0.9983	0.9994	0.9995	0.9996
**b10 Volume**	0.9917	0.9852	0.9983	1.0000	0.9987	0.9987	0.9989
**b200 Volume**	0.9929	0.9811	0.9994	0.9987	1.0000	0.9995	0.9996
**b800 Volume**	0.9939	0.9841	0.9995	0.9987	0.9995	1.0000	0.9999
**ADC Volume**	0.9938	0.9846	0.9996	0.9989	0.9996	0.9999	1.0000

Diameter correlation

	TE75 Diameter	TE300 Diameter	b0 Diameter	b10 Diameter	b200 Diameter	b800 Diameter	ADC Diameter
**TE75 Diameter**	1.0000	0.9695	0.9768	0.9803	0.9777	0.9761	0.9771
**TE300 Diameter**	0.9695	1.0000	0.9549	0.9580	0.9569	0.9538	0.9590
**b0 Diameter**	0.9768	0.9549	1.0000	0.9932	0.9885	0.9745	0.9897
**b10 Diameter**	0.9803	0.9580	0.9932	1.0000	0.9927	0.9793	0.9949
**b200 Diameter**	0.9777	0.9569	0.9885	0.9927	1.0000	0.9813	0.9936
**b800 Diameter**	0.9761	0.9538	0.9745	0.9793	0.9813	1.0000	0.9963
**ADC Diameter**	0.9771	0.9590	0.9897	0.9949	0.9936	0.9963	1.0000

S2. Composition of the radiomic signature per sequence

Radiomic signature T2W TE75

	**Feature**
1	Medium_wavelet-HLH_firstorder_Median
2	Medium_lbp-3D-k_glszm_LowGrayLevelZoneEmphasis
3	Coarse_logarithm_gldm_SmallDependenceHighGrayLevelEmphasis
4	Coarse_lbp-3D-m2_firstorder_Minimum
5	Coarse_lbp-3D-m1_firstorder_Minimum
6	Coarse_exponential_glszm_LowGrayLevelZoneEmphasis
7	Coarse_exponential_glszm_LargeAreaLowGrayLevelEmphasis
8	Coarse_exponential_glszm_GrayLevelNonUniformityNormalized

Radiomic signature T2W TE300

	**Feature**
1	Coarse_lbp-3D-k_glcm_InverseVariance
2	Medium_wavelet-LLL_ngtdm_Busyness
3	Medium_wavelet-HHH_glcm_Idn
4	Medium_square_glcm_JointEnergy
5	Medium_log-sigma-4-0-mm-3D_glszm_SmallAreaLowGrayLevelEmphasis
6	Medium_lbp-3D-k_glrlm_ShortRunLowGrayLevelEmphasis
7	Medium_lbp-3D-k_glrlm_LowGrayLevelRunEmphasis
8	Medium_lbp-3D-k_firstorder_Skewness
9	Coarse_wavelet-HLH_firstorder_Skewness
10	Coarse_wavelet-HHH_glszm_SizeZoneNonUniformityNormalized
11	Coarse_squareroot_glrlm_LongRunEmphasis
12	Coarse_gradient_ngtdm_Coarseness
13	Coarse_original_glrlm_LowGrayLevelRunEmphasis
14	Coarse_original_glrlm_LongRunLowGrayLevelEmphasis
15	Coarse_original_gldm_LowGrayLevelEmphasis
16	Coarse_original_firstorder_RobustMeanAbsoluteDeviation
17	Coarse_log-sigma-1-0-mm-3D_glrlm_ShortRunLowGrayLevelEmphasis
18	Coarse_lbp-3D-k_glszm_SizeZoneNonUniformityNormalized
19	Coarse_lbp-3D-k_glrlm_ShortRunEmphasis
20	Coarse_lbp-3D-k_glrlm_RunPercentage

Radiomic signature b0

	Feature
1	Medium_log-sigma-2-0-mm-3D_firstorder_90Percentile
2	Medium_wavelet-LLH_glcm_Correlation
3	Medium_square_firstorder_Skewness
4	Medium_square_firstorder_Kurtosis
5	Medium_original_firstorder_Skewness
6	Medium_log-sigma-4-0-mm-3D_firstorder_Skewness
7	Medium_lbp-3D-m2_glszm_SmallAreaHighGrayLevelEmphasis
8	Medium_lbp-3D-m2_glszm_HighGrayLevelZoneEmphasis
9	Medium_lbp-3D-m2_glrlm_ShortRunHighGrayLevelEmphasis
10	Medium_lbp-3D-m2_glrlm_LongRunHighGrayLevelEmphasis
11	Medium_lbp-3D-m2_glrlm_HighGrayLevelRunEmphasis
12	Medium_lbp-3D-m2_gldm_HighGrayLevelEmphasis
13	Medium_lbp-3D-m2_glcm_JointAverage
14	Medium_lbp-3D-m2_glcm_Correlation
15	Medium_lbp-3D-m2_glcm_Autocorrelation
16	Medium_lbp-3D-k_glszm_SizeZoneNonUniformityNormalized
17	Medium_lbp-3D-k_glcm_Idmn
18	Medium_lbp-3D-k_firstorder_Range
19	Coarse_wavelet-LHL_glcm_Correlation
20	Coarse_wavelet-HLL_ngtdm_Contrast
21	Coarse_logarithm_glszm_LargeAreaLowGrayLevelEmphasis
22	Coarse_log-sigma-1-0-mm-3D_ngtdm_Strength
23	Coarse_lbp-3D-k_glrlm_ShortRunLowGrayLevelEmphasis

24	Coarse_exponential_glcm_Correlation

Radiomic signature b10

	Feature
1	Coarse_original_firstorder_Skewness
2	Medium_wavelet-HHH_glszm_SmallAreaLowGrayLevelEmphasis
3	Medium_log-sigma-5-0-mm-3D_firstorder_Skewness
4	Medium_log-sigma-4-0-mm-3D_firstorder_Skewness
5	Medium_lbp-3D-m2_gldm_SmallDependenceHighGrayLevelEmphasis
6	Coarse_wavelet-HHL_firstorder_RootMeanSquared
7	Coarse_wavelet-HHL_firstorder_Mean
8	Coarse_log-sigma-2-0-mm-3D_glszm_LowGrayLevelZoneEmphasis
9	Coarse_log-sigma-2-0-mm-3D_glrlm_ShortRunLowGrayLevelEmphasis
10	Coarse_log-sigma-2-0-mm-3D_firstorder_Mean
11	Coarse_lbp-3D-k_ngtdm_Busyness
12	Coarse_exponential_glszm_LowGrayLevelZoneEmphasis
13	Coarse_exponential_glszm_GrayLevelNonUniformityNormalized

Radiomic signature b200

	Feature
1	Coarse_log-sigma-2-0-mm-3D_glcm_JointEnergy
2	Coarse_exponential_glszm_SmallAreaEmphasis
3	Medium_wavelet-LHL_glcm_DifferenceVariance
4	Medium_wavelet-HLH_glcm_Idmn
5	Medium_log-sigma-1-0-mm-3D_firstorder_90Percentile
6	Coarse_wavelet-LLL_firstorder_Skewness
7	Coarse_wavelet-HLL_gldm_LargeDependenceHighGrayLevelEmphasis
8	Coarse_log-sigma-4-0-mm-3D_glszm_SmallAreaEmphasis
9	Coarse_log-sigma-3-0-mm-3D_glszm_SmallAreaEmphasis
10	Coarse_log-sigma-3-0-mm-3D_glcm_DifferenceVariance
11	Coarse_log-sigma-3-0-mm-3D_firstorder_Kurtosis
12	Coarse_log-sigma-2-0-mm-3D_glszm_HighGrayLevelZoneEmphasis
13	Coarse_log-sigma-2-0-mm-3D_glcm_SumSquares
14	Coarse_log-sigma-1-0-mm-3D_glrlm_ShortRunHighGrayLevelEmphasis
15	Coarse_gradient_firstorder_Skewness
16	Coarse_exponential_glszm_LowGrayLevelZoneEmphasis
17	Coarse_exponential_glrlm_ShortRunLowGrayLevelEmphasis

Radiomic signature b800

	**Feature**
1	Coarse_log-sigma-2-0-mm-3D_gldm_LowGrayLevelEmphasis
2	Medium_lbp-3D-m2_gldm_SmallDependenceHighGrayLevelEmphasis
3	Medium_lbp-3D-m2_firstorder_Range
4	Coarse_log-sigma-3-0-mm-3D_glrlm_ShortRunLowGrayLevelEmphasis
5	Coarse_log-sigma-3-0-mm-3D_glrlm_LowGrayLevelRunEmphasis
6	Coarse_log-sigma-3-0-mm-3D_gldm_LowGrayLevelEmphasis
7	Coarse_log-sigma-2-0-mm-3D_glrlm_LowGrayLevelRunEmphasis

Radiomic signature ADC

	**Feature**
1	Medium_log-sigma-5-0-mm-3D_firstorder_Skewness
2	Medium_log-sigma-4-0-mm-3D_firstorder_Skewness
3	Medium_lbp-3D-m1_glcm_ClusterTendency
4	Coarse_wavelet-LLL_firstorder_Skewness
5	Coarse_wavelet-LLH_glcm_Idmn
6	Coarse_wavelet-LHL_glcm_Correlation
7	Coarse_wavelet-LHL_firstorder_Skewness
8	Coarse_wavelet-HLL_glcm_Correlation
9	Coarse_wavelet-HHL_gldm_DependenceNonUniformityNormalized
10	Coarse_wavelet-HHL_glcm_Correlation
11	Coarse_log-sigma-5-0-mm-3D_glcm_ClusterShade
12	Coarse_log-sigma-5-0-mm-3D_firstorder_Skewness

S3. HIF-1 alpha staining protocol

Immunohistochemistry of the Formalin-Fixed Paraffin-Embedded (FFPE) tumour samples was performed on a BenchMark Ultra autostainer (Ventana Medical Systems). Paraffin sections were cut at 3 µm, heated at 75°C for 28 minutes and deparaffinized in the instrument with an EZ prep solution (Ventana Medical Systems). Heat-induced antigen retrieval was carried out using Cell Conditioning 1 (CC1, Ventana Medical Systems) for 64 minutes at 95°C. HIF-1 alpha was detected using clone 54/HIF-1a (1/50 %dilution, 64 minutes at 36°C, BD Transduction Laboratories), OptiView DAB Detection Kit (Ventana Medical Systems). Slides were counterstained with Hematoxylin II and Bluing Reagent (Ventana Medical Systems). From each slide, a high-resolution digital scan was obtained and automatically pseudonymized with study-specific identifiers and subsequently uploaded to Slide Score, a cross-platform web application that facilitates the scoring of whole slide images by multiple pathologists (https://www.slidescore.com/).

S4. Selected machine learning models/pipelines per sequence. For pipelines where more than one model is mentioned, stacking was used to combine the classifiers.

Sequence	Machine learning pipeline
T2 TE75	K Nearest Neighbours
T2 TE300	Logistic Regression with Gaussian Naive Bayes
DWI b0	Decision Tree with Gaussian Naive Bayes
DWI b10	K Nearest Neighbours
DWI b200	Logistic Regression with Decision Tree
DWI b800	Decision Tree with Bernoulli Naive Bayes
DWI ADC	Gaussian Naive Bayes with Logistic Regression
